# Goose management in Scotland: An overview

**DOI:** 10.1007/s13280-016-0883-5

**Published:** 2017-02-18

**Authors:** Ian Bainbridge

**Affiliations:** Luckie Harg’s, Anwoth, Gatehouse of Fleet, Castle Douglas, DG7 2EF Scotland, UK

**Keywords:** Adaptive management, Goose populations, Wildlife policy

## Abstract

This paper reviews the changing status of goose populations in Scotland since the 1940s, and the changing policies and management activities employed over that time. The size of all goose populations has risen since the 1940s due to protection measures, changes in agriculture and levels of shooting, in the UK and elsewhere. The development of goose policies in response to public interest and pressures is described. Some changes in goose populations since 2000 can be linked to this policy development. Policy is now split between protective measures for some species and adaptive management approaches encompassing control measures for others. The paper identifies the importance of the social and economic concerns of some parties in the development of goose management approaches, rather than scientific advice based on goose population numbers and trends, and recognises that future goose policy will necessarily be a difficult compromise between wide ranging, and even opposing positions and views.

## Introduction: Scottish goose populations in the twentieth century

In the 1940s, wild geese were rare in Scotland. Of the nine populations of geese found in Scotland (Table 1), only the Icelandic=breeding population of greylag geese *Anser anser* exceeded 10 000 birds. As systematic counting of goose populations only began in the 1950s, the picture before then is unclear, but it is highly likely that a combination of hunting for food and sport, systematic persecution and the disruption of the Second World War combined to deplete most of the goose populations. The Solway population of barnacle geese *Branta leucopsis* was considered to have reached a low of around 300 birds in the mid-1940s, the nadir of the Icelandic pink-footed goose *Anser brachyrhynchus* population may have been 5000 birds, and Greenland white-fronted geese *Anser albifrons flavirostris* may have numbered around 2000 at that time. Scottish-resident greylag geese were persecuted almost to extinction by farmers and crofters, essentially becoming limited to South Uist in the Western Isles, with a few birds in remote parts of Caithness and Sutherland (Kirby et al. 1999).

**Table 1:** Changes in Scottish Goose population abundance, 1948–2015

Population	ca. 1950 numbers	ca. 2000 numbers	ca. 2015 numbers
Svalbard barnacle goose *Branta leucopsis*	300 (1948)	24 000 (1999)	38 100 (2013/2014)
Greenland barnacle goose *Branta leucopsis*	8080 (1959)	53 823 (1999)	80 670 (2013)
Greenland white-fronted goose *Anser albifrons flavirostris*	3000-4000 (1950s)	21 997 (1999)	8558 (2015)
Pink-footed goose *Anser brachyrhynchus*	49 700 (1957)	245 349 (2000/01)	393 170 (2015)
Icelandic greylag goose *Anser anser*	25 000 (1952)	80 324 (2000/2001)	89 668 (2015)
Scottish native greylag goose *Anser anser*	100 pairs? (1950s)	10 000 (1997)	46 400 (2015)
Reintroduced greylag goose *Anser anser*	2000? (1950s)	2673 (1991)	12 895 (2008/2009)
Taiga Bean goose *Anser fabalis fabalis*	200 (1952)	180 (2000/01)	231 (2014)
Light-bellied Brent goose *Branta bernicla hrota*	0? (1950s)	5–10 (2000)	140 (2015)
Canada Goose *Branta canadensis*	119–194 (1953)	1244 (2000)	3000+? (2015)
Snow Goose *Anser caerulescens*	Introduced to Mull	34 (2002)	20–40 (2014)

*Sources* *ca. 1950s* Boyd (1963), Ogilvie (1969), Kirby et al. (1999); *ca. 2000* summary from Crabtree et al. (2010), Scottish Bird reports on line, Fox and Francis (1999); *ca. 2015*: summary from Cohen (2015), Fox et al. (2015)

During the 1940s, ornithologists recognised the perilous state of Scotland’s goose populations, and the Protection of Birds Act 1954 was an important first step in providing protection to them. From the late 1940s (and systematically recorded since the 1950s), there was a steady increase in most of the goose populations. This has been attributed to increased protection, a decline in the persecution of geese by farmers, a response by geese to modern intensive farming methods, voluntary limits on shooting and other factors outside the UK. For example, Greenland barnacle goose numbers on Islay rose from around 2000 in 1952 to 40 000 in 2003 (Crabtree et al. 2010), while Svalbard barnacle geese on the Solway rose from a low of 300 birds in 1948 to around 23 000 in 1997, thanks to the protection both in Scotland in 1954 and in Svalbard in 1955 (Kirby et al. 1999).

Over much of the same period, Icelandic greylag and pink-footed goose populations followed a similar trajectory. Icelandic greylag numbers rose from 20 000 in the 1950s to around 80 000 in the 1970s, after which the population stabilised. In contrast, pink-footed goose numbers continued to rise after the 1970s and by the mid-1990s had reached around 225 000 birds. Greenland white-fronted goose numbers rose more slowly, from 3000 to 4000 in the 1960s to a peak of around 22 000 in 1998/1999 (Fox and Francis 1999).

In contrast to most of the Arctic breeding geese, the resident Scottish greylag goose population remained very small and localised; so much so that in 1958, Loch Druidibeg on South Uist was declared a National Nature Reserve “for the largest remaining breeding population of native greylag geese in Britain (65 pairs)” (Ratcliffe 1977). Throughout this time, a number of eggs were taken from the Uists, mostly by shooting estate managers, as a means of reintroducing greylag geese elsewhere in Britain. These actions largely gave rise to the current and growing resident populations of greylag geese south of the Scottish Highlands, as well as on Orkney and other areas of Scotland. The passing of the Wildlife and Countryside Act (1981) gave resident greylag geese further protection during the breeding season in their remaining key breeding areas of the Outer Hebrides, Caithness, Sutherland and Wester Ross.

The Wildlife and Countryside Act confirmed the protection of most goose species, enforcing the EU Birds Directive of 1979 in the UK, and leaving only Canada *Branta canadensis*, greylag and pink-footed geese as species which could be legitimately shot in the ‘open’ hunting season in Scotland. Some populations responded rapidly to this added protection. For example, the Scottish wintering population of Greenland white-fronted geese was around 7200 in 1983 (Stroud 1984), but following protection from hunting effectively from that year, the population grew steadily to peak at just under 22 000 in 1999 (Fox and Francis 1999).

During the 1980s and early 1990s, all of the Scottish goose populations were considered to be increasing, or at least stable, and farming concerns began to rise. An increasing number of complaints were received by the Scottish government^1^ about agricultural damage and loss of yield due to goose grazing on grass and cereal crops. In parallel with this, a number of Special Protection Areas (SPAs)—which were required to be designated and protected under the terms of the European Birds Directive—were established for protected goose species. Four SPAs were established on Islay in 1988, and these gave rise to the first goose management agreements, between individual farmers whose land was within the SPAs and the government nature conservation agency (at the time, the Nature Conservancy Council).^2^

## Development of Scottish National Goose Policy

The 1990s saw a continuing rise in goose numbers, rising agricultural concerns voiced in political circles, and the establishment of a number of ‘goose schemes’, mostly linked to the goose SPAs. These were on Islay (for Greenland barnacle geese and Greenland white-fronted geese), the Solway (Svalbard barnacle geese), South Walls, Orkney (Greenland barnacle geese), the Loch of Strathbeg, Aberdeenshire (pink-footed geese) and the Uists (resident greylag geese). All schemes except the Uists made provision for direct payments for farmers to allow geese to feed undisturbed on certain fields. This gave rise to the first broad public discussions about the future of goose management, and the publication by the Scottish government of ‘Wild Geese and Agriculture in Scotland: a discussion paper’ (Scottish Office 1996). This in turn led to the establishment of the National Goose Forum for Scotland in 1997, which brought together government, statutory and non-government bodies to debate the issues surrounding goose management. The Scottish government also commissioned the Wildfowl and Wetlands Trust (WWT), a UK non-government specialist body, to produce a review of “Geese and their Interactions with Agriculture and the Environment” (Kirby et al. 1999), describing goose populations and trends, the farming systems on which geese feed, methods of management and control, and the potential for modelling of goose population trends and their effects on Scottish agriculture.

The deliberations of the National Goose Forum concluded in 2000 with the publication of a policy report and 34 recommendations (Scottish Executive 2000). This led to the formation of the National Goose Management Review Group (NGMRG) in 2001, whose role, continuing to the present day, was to oversee the implementation of these recommendations, including the development of local goose management schemes to ensure the monitoring of protected goose populations and to conduct quinquennial reviews of goose management (Scottish Executive 2000).

The National Goose Policy objectives for goose management schemes in Scotland, set in 2000 and only subject to minor modifications of wording since, are as follows:

- To meet the UK’s nature conservation obligations for geese, within the context of wider biodiversity objectives.
- To minimise economic losses experienced by farmers and crofters as a result of the presence of geese.
- To maximise the value for money of public expenditure.

In parallel to these developments, Scottish Natural Heritage (SNH) was developing replacement goose management schemes designed to reflect the new policies, and in 2000, Local Goose Management Schemes (LGMS) were established for goose populations at South Walls on Orkney, Islay, Kintyre and the north Solway (Scottish Natural Heritage 2000), all of which were based on payments to allow feeding on some areas, and a range of scaring options in other areas. These have continued relatively unchanged until today. These schemes were aimed at the specially protected species (barnacle and white-fronted geese), especially where they occurred at high densities. The 2000 policy made special provision for sites with SPAs for other species when present in large numbers and high densities, and in 2001 the Loch of Strathbeg LGMS was added, aimed at protecting the large numbers of spring-roosting pink-footed geese on the Loch of Strathbeg SPA.

At its first meeting in 2001, because of a well-recognised need for scientific review and advice, the NGMRG proposed the formation of a Goose Science Advisory Group (GSAG). GSAG comprises scientific staff from Government, agencies and conservation and shooting NGOs. It provides advice to the NGMRG on all goose science issues, reviews and guide censuses and trends information, and advises on population modelling, damage assessments and research projects. Its intent is to provide scientific consensus on goose science, and avoid the political debate which inevitably occurs in the plenary meetings of NGMRG. One of the first activities of GSAG was to consider methods of modelling of future goose population trends, which led to the publication of a series of Population Viability Analyses for the key goose populations (Trinder et al. 2005, 2009; Trinder 2010a, b, 2014a, b).

The NGMRG also commissioned economic appraisals of the costs and benefits of managing geese in Scotland (MacMillan et al. 2001), and established the review processes required by the policy forum. These review processes proposed that the LGMSs should be reviewed annually by the NGMRG; that the Local Scheme financial agreements would be reviewed every 5 years; that there would be an internal review of policy and payments every 5 years and that there would be a full independent review every 10 years, the first of which was undertaken in 2010 (Crabtree et al. 2010).

## The last 10 years

One of the characteristics of goose populations in Scotland over the last 50 years has been unexpected change (Table 1). Why did the Icelandic greylag goose population suddenly stop increasing in the 1980s? Why did Greenland white-fronted geese peak in the late 1990s and experience a steady decline since? Why did the small Taiga bean goose *Anser fabalis fabalis* population suddenly change its wintering location by over 100 km?

The last decade has been no different; we have seen the resident greylag goose population continue its near-exponential rise. This has occurred in both the north Scotland native bird population and the reintroduced population further south. On Orkney alone, the population has risen from a few pairs in 1990, to around 100 pairs in 2000 (Meek 2008), and now to almost 23 000 birds counted in August 2014 (Mitchell et al. 2014). Overall, the resident Scottish greylag population was estimated at 47 500 in 2009 (Mitchell et al. 2010), and has doubtless continued to rise, as the northern and southern populations merge (Mitchell et al. 2012). The biggest change in distribution has been that of the Icelandic greylag goose population, which has all but abandoned central Scotland in winter, with the vast majority (over 60 000) of the population now wintering on Orkney, when 10 years ago, only a few thousand did so (Mitchell 2015). This appears to be a classic case of short-stopping, with the birds exploiting the large number of improved grass fields on Orkney (Mitchell 2015). After decades of increases, the Islay barnacle goose population appears to have reached a plateau, and may even be declining, while the smaller Greenland barnacle goose populations elsewhere continue to increase (Mitchell and Hall 2013). In this period, the Svalbard barnacle goose population has continued to rise steadily and in 2013–14 reached 38 100 birds (WWT 2014). Greenland white-fronted geese have continued to decline through this period, down to around 8600 in Scotland in spring 2015, less than 50% of their peak population level (Fox et al. 2015). Bean goose numbers have increased slowly in Scotland (Minshull 2016), and a new SPA was declared in 2008, covering their main wintering area. This is in contrast to the only other UK bean goose population in Norfolk, whose numbers have declined throughout the last 10 years.

Political and social circumstances have also continued to change. As most Local Goose Management Schemes had payments based on goose densities, rising goose numbers resulted in higher costs to government. Over the last 10 years, the Scottish government has made clear that goose payments are not intended to cover all agricultural costs and losses, and has sought to cap costs overall. These tighter finances have given rise to increased dissatisfaction and more political lobbying by some farmers’ groups, and renewed calls for management of goose numbers, notably of resident greylag geese on the Uists, Tiree & Coll and Orkney and of Greenland barnacle geese on Islay. There has been substantial local pressure for new Local Goose Management Schemes on Orkney, for both resident and wintering greylag geese, and for resident greylag goose schemes elsewhere, but the Scottish government has declined to establish any new payment schemes for goose populations which are not specially protected. Instead, schemes were established on The Uists, Coll and Tiree, and Orkney in 2009 which paid for professional goose scaring by shooting, notably in areas with resident greylags, but rising numbers and continued political pressure gave rise to the development of ideas to limit the size of local resident greylag goose populations. Comparisons have been made with the approach to the management of deer populations in Scotland, where it has long been accepted that numbers (or densities) should be limited in a particular area. This is in contrast to the British approach to the management of bird populations, which has been not to intervene, but to let numbers limit themselves (Stroud et al. 2016).

## Adaptive management pilot schemes

In response to these changes, SNH was asked by the Scottish government to develop an approach for the introduction of adaptive management schemes for some island populations of resident greylag geese. These were termed ‘Pilot’ schemes in that they would be time-limited, with the goal of self-help once numbers were at a manageable level, and that they would test whether an adaptive management approach (Williams et al. 2009) could be adopted in Scotland. This resulted in schemes being launched in 2012 on Orkney, the Uists, Tiree and Coll and in 2015 on Lewis and Harris. In contrast to most migrant goose populations, demographic data on resident greylag populations, especially at the level of an island group, were lacking. A population viability analysis for resident greylag geese, based largely on data from the Uists (Trinder et al. 2009) gave some guidance, but could not account for local circumstances. SNH, working with the local interested parties, proposed a concept that managed population sizes should be based on an approximate target density of geese on the available improved grassland on each island group, with densities set at lower levels for those island areas which also received large numbers of migrant wintering geese. In the absence of detailed population data, simple deterministic models were developed. These models used count and simple productivity data to estimate the shooting levels required to achieve a population trajectory towards the desired number of birds over a five- to ten-year period. This model would be rerun annually, using the latest annual count and productivity data (collected as part of the scheme), to identify the required shooting levels for the coming year, which were set in the early autumn. Licensed shooting would then take place in early autumn, followed by further shooting in the normal open hunting season from October to January. It is too early to say whether these pilot schemes have been successful: numbers do appear to have been limited and reduced on Tiree & Coll and Uist. On Orkney, the rapid increase in numbers appears to have been halted, but numbers have not fallen, perhaps because estimates suggest that as many as 8000 resident geese may need to be shot annually to have a significant effect in reducing the population below its current level of around 23 000 birds. Shooting at this level, much of which needs to occur in the limited time period before Icelandic migrants arrive in October, may be beyond the capacity of the local shooting community, and other new approaches to meeting shooting targets may be necessary.

As the greylag pilots were being developed, calls by farmers on Islay for the management of barnacle goose numbers were growing. Although the numbers of barnacle geese were no longer rising, the cap on funding the scheme gave rise to new and effective political lobbying. Although the geese provide substantial income to the island from wildlife tourism, the Scottish government took the view that further action was necessary, and asked SNH to develop an Islay Sustainable Goose Management Strategy, to provide extra conservation measures for the declining population of Greenland white-fronted geese, and also to reduce agricultural damage by limiting barnacle goose numbers. The detail of this scheme is provided in McKenzie and Shaw (2017). This proposal crossed another red line with its unprecedented and controversial call for population management of a bird species specially protected by its listing on Annex 1 of the Birds Directive.

To summarise, the current position of goose management in Scotland is that there are a limited number of winter schemes based on payments to allow feeding of geese on agricultural land. There is a limited shooting associated with these schemes (Islay only), and a general trend to decreasing levels of funding; with a resulting increase in farmer dissatisfaction and political activity. The emphasis on goose management policy remains on specially protected goose populations. There are also localised summer/autumn pilot schemes for resident greylag geese based on adaptive population management, and there is a move towards adaptive management for barnacle geese on Islay.

## Some dichotomies

The development of the current suite of goose schemes and their management activities are clearly not based on goose numbers or demographies alone. The levels of payment agreed for each scheme depend in part on the farming systems, the remoteness of the locations, the numbers of geese involved in the scheme and the methods of calculating payments. However, no combination of the demographic and economic data can explain the differences in management between schemes, and simple comparisons between the goose populations and their management show clearly that much of the decision-making has been of a socio-political nature, responding to the strength of local concerns. This mirrors the development of the Flyway Management Plan for Svalbard-breeding pink-footed geese (Madsen and Williams 2012), which was developed in response to farmers’ concerns, especially in Norway.

The most numerous goose population in Scotland, the Icelandic pink-footed goose, whose population is approaching 400 000 birds, is among the least problematic, and those goose populations receiving the most drastic interventions are amongst the smallest. Three simple comparisons are made in Table 2 to illustrate the differences and dichotomies of approaches to the management of goose populations. In many respects, the Islay and Solway barnacle goose populations are very similar, but are subject to increasingly different interventions. With Icelandic greylag and pink-footed geese, the latter species, which is far more numerous, elicits far less concern and receives far less attention, because it often feeds on spent crops and has a nomadic winter life. In the case of native and reintroduced greylag geese in Scotland, there are many similarities in their increasing population trends but very different reactions to their management.

**Table 2:** Comparisons between goose populations in Scotland and their management

*Islay (Greenland) barnacle geese*	*Solway (Svalbard) barnacle geese*
Static numbers: ca. 37 000	Rising numbers: ca. 37 000
Large management payments	Modest management payments
Feeding scheme	Feeding scheme
Move to adaptive management	No shooting agreed by local scheme
Shooting controls	Small political activity
Strong political activity	*Icelandic pink-footed geese*
*Icelandic greylag geese*	Rising numbers: ca. 390 000
Static numbers: ca. 90 000	Highly mobile, daily and seasonally
Concentrated geographically on Orkney	Very small payment scheme - Strathbeg
No payment schemes	Almost no political activity
Modest (increasing) political activity	Open season shooting
Open season shooting	*Reintroduced resident greylag geese*
*Native greylag geese*	Rising numbers: 30 000?
Rising numbers: ca. 40 000	Widespread, locally very numerous
Concentrated geographically	No payments
Small payments for shooting schemes	Almost no political activity
Strong political activity	No management
Four local Adaptive Management Pilot schemes	Open season shooting
Shooting controls and open season shooting	

The reflection on this is that the goose numbers do not predict, drive or control approaches to goose management, nor do their population demographies or trajectories. There may be a link to goose populations being concentrated or resident in a relatively small area, perhaps especially on improved grassland or growing crops. However, it is clear that the socio-political aspects, the local concerns and the levels of political activity resulting from these play a strong role in the actions that follow and in the development of local goose schemes.

## Final thoughts

Over twenty-five years of debate, argument, research and policy have demonstrated that goose management in Scotland has been neither simple nor comfortable. Unexpected changes in goose numbers and behaviour, agriculture and politics have added to the complications, and it would be a good motto for those working on goose management in future to “expect the unexpected”. In many parts of the world, conservation activities have led to major ongoing increases in goose populations which a few decades ago were under real threat of extinction, but these examples all point to the fact that it would be unwise to maintain a non-interventionist approach for the future. The political reality of many cases is that we need to adopt an adaptive management approach (Williams et al. 2009) which recognises the legitimate interest of a wide range of parties, from farmers to conservationists, from hunters to eco-tourism businesses, and we must involve all parties in working out what to do. Science can only contribute to a solution by providing sound evidence on which to base management actions, monitoring the effects of those actions and helping to refine subsequent management. It will help us all to understand the political processes involved, and to recognise that human reactions and perceptions are not based on scientific logic. Most of all, we need to recognise that management actions and solutions are highly unlikely to represent the perfect outcome from any individual perspective, and that all parties will need to be prepared to compromise to reach a mutually acceptable approach. Perhaps we should be looking for the point of minimum mutual unhappiness for all the parties, where no one is entirely satisfied but everyone is prepared to accept compromises which work towards solutions.

## Footnotes

1 Throughout this period, the governance of Scotland has changed substantially, with devolution of many powers from the UK government. The government has been called The Scottish Office, Scottish Executive and Scottish Government at various times, but for the sake of simplicity it is termed Scottish government throughout this paper.

2 Similarly, the government agency responsible for nature conservation has changed over the time period. The Nature Conservancy, which became the Nature Conservancy Council (NCC) in 1973, was devolved into NCC Scotland in 1991, and then became Scottish Natural Heritage in 1992.
